# Plasma Cell Gingivitis: Clinical Presentation, Histopathologic Correlation, and Therapeutic Challenges

**DOI:** 10.3390/clinpract15090158

**Published:** 2025-08-28

**Authors:** Davide Gerardi, Diana Torge, Sara Bernardi, Pierangelo Burdo, Maurizio Piattelli, Giuseppe Varvara

**Affiliations:** 1 Department of Life, Health and Environmental Sciences, University of L’Aquila, 67100 L’Aquila, Italy; davide.gerardi@graduate.univaq.it (D.G.); sara.bernardi@univaq.it (S.B.); 2 Department of Innovative Technologies in Medicine & Dentistry, University “G. d’Annunzio” of Chieti-Pescara, 66100 Chieti, Italy; pierangeloburdo@libero.it (P.B.); mpiattel@unich.it (M.P.); gvarvara@unich.it (G.V.); 3 Department of Life Science, Health, and Health Professions, Link Campus University, 00165 Rome, Italy

**Keywords:** plasma cell gingivitis, case report, immunohistochemistry, topical corticosteroid therapy

## Abstract

**Background/Objectives:** Plasma cell gingivitis (PCG) is a rare, benign, non-dental-plaque-induced inflammatory condition characterized by dense subepithelial infiltration of polyclonal plasma cells. Due to its nonspecific clinical presentation, PCG represents a diagnostic challenge. This case report aims to describe a clinical case of PCG, highlighting the diagnostic process, histopathological correlation, and therapeutic approach. **Methods:** A 57-year-old male presented with a polypoid, erythematous, and edematous gingival lesion in the anterior maxillary region, with spontaneous bleeding on probing. Following clinic assessment, an incisional biopsy was performed, alongside complete hematological and inflammatory profiling. Histological and immunohistochemical analyses revealed the presence of an inflammatory infiltrate. **Results:** Histological evaluation revealed spongiotic squamous epithelium characterized by a dense plasma cell infiltrate with a liquenoid pattern of CD3-positive T and CD20-positive B lymphocytes. A polytypic expression of kappa and lambda light chains was also detected. The patient underwent topical corticosteroid therapy, showing progressive clinical improvement and resolution of symptoms, although minor mucosal involvement persisted. **Conclusions:** PCG remains a rare and underdiagnosed condition requiring integration of clinical, hematological, and histopathological data for accurate diagnosis. While corticosteroids remain the first-line therapy, emerging treatments, including photobiomodulation, may offer future adjunctive strategies to improve outcomes and reduce recurrence.

## 1. Introduction

Plasma cell gingivitis (PCG) is a rare, benign, non-dental-plaque-induced inflammatory condition of the gingiva characterized by dense polyclonal plasma cell infiltration in the subepithelial connective tissue [1]. This benign inflammatory condition of the gingiva has been described in literature under various synonyms: PCG, idiopathic gingivostomatitis, plasma cell gingivostomatitis, atypical gingivostomatitis, allergic gingivostomatitis, and gingival plasmacytosis [2]. The recent periodontal classification includes PCG in the macro group of gingival diseases—non-dental-plaque-induced, as an inflammatory and immune condition, determined by a hypersensitivity reaction [3].

The incidence of this pathology is very low: a recent systematic review conducted by Atarbashi-Moghadam et al. [4] of only confirmed PCG cases reported 13 female and 6 male patients (19 total) aged 9–64 years. All the cases involved the anterior maxilla, associated with bleeding and swelling; diagnosis was based on microscopic evaluation, and a variety of treatment protocols were used, ranging from lesion removal to photobiomodulation [4]. Other studies, such as Leuci et al. [1], have reported larger case series but did not meet the inclusion criteria of the review [1].

The exact etiopathogenesis of PCG remains unknown. It is generally considered a hypersensitivity reaction of the gingival tissues to an external antigen, although in most patients these lesions appear to be idiopathic [5]. From a pathogenetic perspective, chronic inflammation may contribute to local immunological dysregulation, promoting the recruitment of plasma cells. Pro-inflammatory cytokines appear to play a central role in mediating this process by driving B-cell activation and proliferation within the affected tissues [1].

This benign pathology involving the gingiva displays nonspecific clinical signs, probably associated with an erythematous gingiva exhibiting an irregular surface and soft tissue edema, with loss of normal gingival architecture, which may extend to the mucogingival junction [2]. The main symptoms reported by patients are bleeding on brushing or discomfort caused by the gingival swelling [6].

In rare instances, PCG is accompanied by plasma cell inflammation in other oral sites—for example, concurrent cheilitis or glossitis has been observed in a subset of patients, forming part of a triad of plasma cell gingivitis, cheilitis, and glossitis [7,8]. Such cases are sometimes considered under the broader spectrum of “plasma cell mucositis,” wherein multiple oral mucosal sites are affected by a similar plasma cell infiltrative process [9].

Due to its common characteristics, plasma cell gingivitis should be considered in the differential diagnosis of gingival manifestations of systemic diseases, such as acute leukemia or multiple myeloma, or extraosseous manifestations of solitary plasmacytoma of the jaws [10]. Thus, a thorough evaluation is mandatory. The diagnosis of PCG involves hematological screening in addition to clinical and histopathological examinations [8].

This study reports a recent case report of the previously described rare pathology, highlighting its diagnostic and treatment challenges.

## 2. Case-Report

A 57-year-old male patient was referred to the Dental Clinic of the University “G. d’Annunzio” in Chieti-Pescara reporting discomfort due to the presence of a swollen area in the anterior region extending to the premolars of the upper maxilla, along with bleeding during brushing.

The patient’s medical history was clear, with no systemic or local conditions reported. No relevant family history or current pharmacological treatments were noted. The patient denied allergies, smoking, or alcohol use. Dental history was non-contributory.

**Figure 1:**
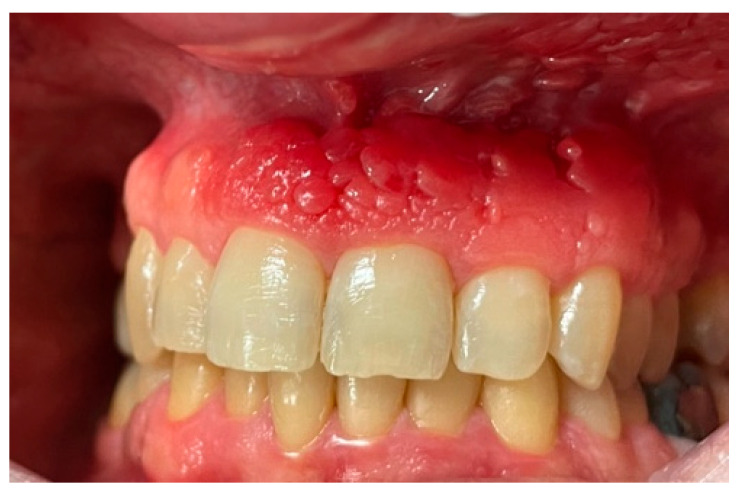
Clinical presentation of the lesion at the first clinical assessment: polypoid, erythematous, and edematous gingival tissue localized in the anterior maxillary region, with spontaneous bleeding upon probing.

**Figure 2:**
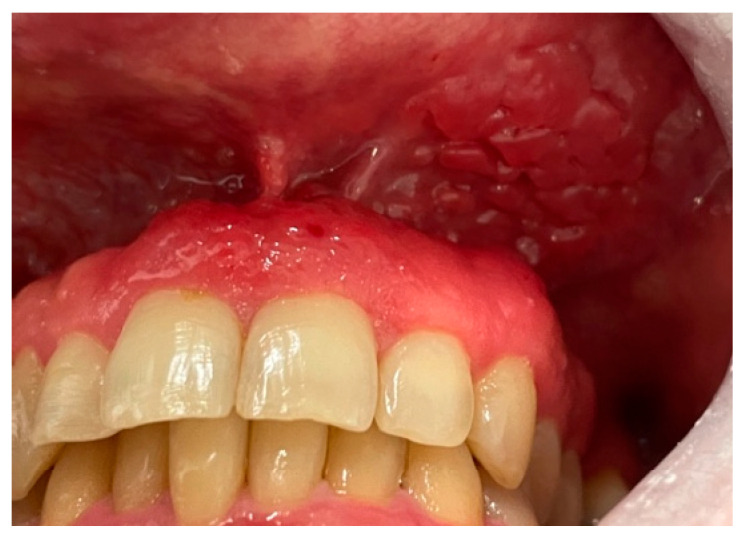
Close-up view showing the extension of the lesion involving the marginal and attached gingiva, as well as the adjacent oral mucosa, with an irregular surface texture and loss of stippling.

Intraoral examination revealed an erythematous and edematous lesion in the maxillary region, extending from the upper right central incisor to the upper left canine, involving the adjacent alveolar mucosa. The gingiva appeared polypoid and bled upon probing. The gingival tissues in the adjacent areas were clinically healthy, with only mild inflammation, likely secondary to suboptimal oral hygiene (Figure 1 and Figure 2).

Based on the initial clinical assessment, the lesion was provisionally diagnosed as an inflammatory hyperplastic reaction involving the gingiva and alveolar mucosa; therefore, a session of professional oral hygiene was prescribed, along with the use of a chlorhexidine-based mouthwash twice a day until the date of the next appointment. A 14-day follow-up was scheduled to monitor the lesion and plan the biopsy to confirm the diagnosis [4].

### 2.1. Treatment Plan

The patient was scheduled for follow-up and incisional biopsy after fourteen days, and oral hygiene instructions were provided.

At the 14-day follow-up, an incisional biopsy was performed at two distinct sites: one at the level of the keratinized paramarginal gingiva, and the other at the alveolar mucosa. The biopsy sites were closed using 4/0 absorbable sutures.

In addition, blood tests, including complete blood count with differential, reticulocyte count, erythrocyte sedimentation rate (ESR), and quantitative C-reactive protein (CRP) assay, were prescribed to comprehensively assess systemic inflammation and exclude any potential systemic involvement of the underlying pathology.

The analysis demonstrated a mildly elevated eosinophil percentage (Eos%) (Table 1).

### 2.2. Histopathological and Immunohistochemical Examination

**Figure 3:**
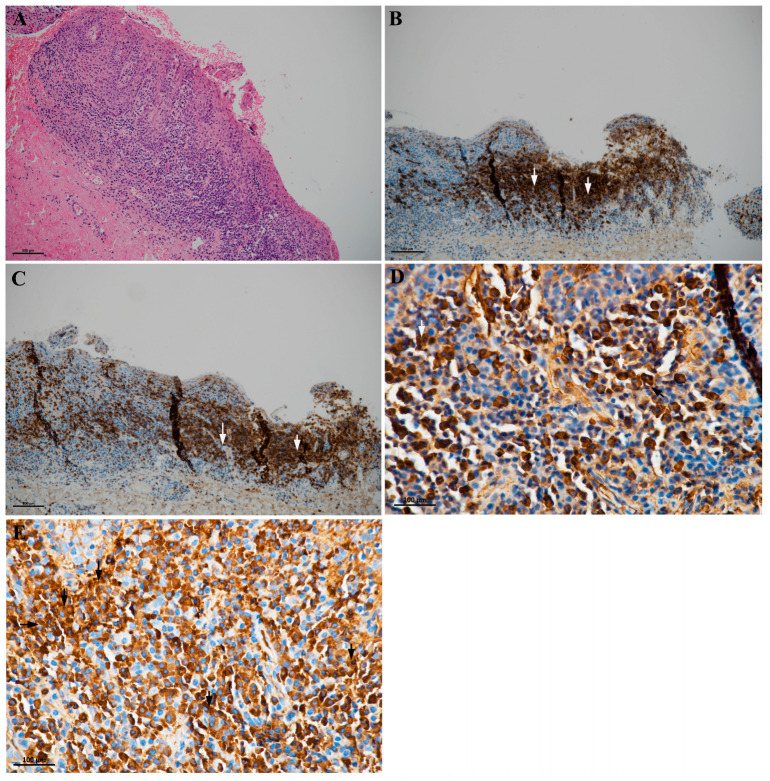
Histopathological examination of the oral mucosa: LM representative images. (**A**) LM images of case depicting a thickened and spongiotic squamous epithelium with dense plasma cell infiltrate, with a liquenoid pattern (Magnification: 10×; Scale Bar: 500 μm). (**B**) CD20-positive B lymphocytes (white arrows) in the oral mucosa with mixed infiltrate (Magnification: 10×; Scale Bar: 500 μm). (**C**) LM photographs of case depicting mucosa of the oral cavity with fibrosis of the subepithelial connective tissue, intense lichenoid-type infiltrate with CD3-positive T lymphocytes (white arrows) (Magnification: 10×; Scale Bar: 500 μm). (**D**) Numerous plasma cells with polytypic expression of immunoglobulin lambda light chains (white arrows) and kappa light chains (black arrows) were reported (Magnification: 40×; Scale Bar: 100 μm). (**E**) Mucosa of the oral cavity of case depicting the presence of plasma cells, with hyperchromatic and eccentrically placed nuclei, and characterized by the expression of immunoglobulin kappa light chains (black arrows) (Magnification: 40×; Scale Bar: 100 μm).

Histopathological observations, performed using a phase-contrast light microscope (ZEISS Primovert, Jena, Germany), revealed a thickened, spongiotic squamous epithelium characterized by a dense plasma cell infiltrate with a liquenoid pattern in the oral mucosa (Figure 3A). The underlying connective tissue stroma revealed fibrocellular tissue, infiltrated with chronic inflammatory cells: the infiltrate consisted predominantly of plasma cells and lymphocytes. The dense lymphocytic infiltrate was primarily characterized by the following immunophenotypes: B and T lymphocytes (Figure 3B,C). Finally, juxtaepithelial tissues displayed a dense inflammatory infiltrate, rich in mature plasma cells. Furthermore, histochemical and immunohistochemical evaluations included the following markers: PAS, CD20 (Figure 3B), CD3 (Figure 3C), Ki-67, p16, and immunoglobulin lambda and kappa light chains, to confirm and define the plasma cell population (Figure 3D,E). Specifically, immunohistochemical examinations revealed the presence of a mixed infiltrate, with CD20-positive B lymphocytes (Figure 3B). Additionally, light microscopy (LM) photographs of this case depicted the mucosa of the oral cavity, characterized by fibrosis of the subepithelial connective tissue and intense lichenoid-type infiltrate with CD3-positive T lymphocytes (Figure 3C). Finally, at higher magnification, several plasma cells displayed hyperchromatic and eccentrically placed nuclei (“cartwheel-shaped nucleus”) and were characterized by polytypic expression of immunoglobulin lambda (Figure 3D) and kappa (Figure 3D,E) light chains.

### 2.3. Pharmacological Management Protocol for Plasma Cell Gingivitis

The patient was instructed to apply clobetasol propionate 0.05%, compounded in a hydroxypropyl methylcellulose oral base (Biotene Gel), as a bioadhesive and protective vehicle, directly to the affected gingival areas. A small amount of the preparation (approximately pea-sized) was applied three times daily, preferably after meals and before bedtime, following routine oral hygiene practices. The patient was advised to avoid eating, drinking, or rinsing for at least 30 min following each application to ensure optimal mucosal adherence. The treatment was administered for four consecutive weeks. An additional course in pharmacological treatment, as prescribed, would be considered necessary based on clinical judgment, provided that no other complications of a different nature have arisen.

### 2.4. Follow-Up

**Figure 4:**
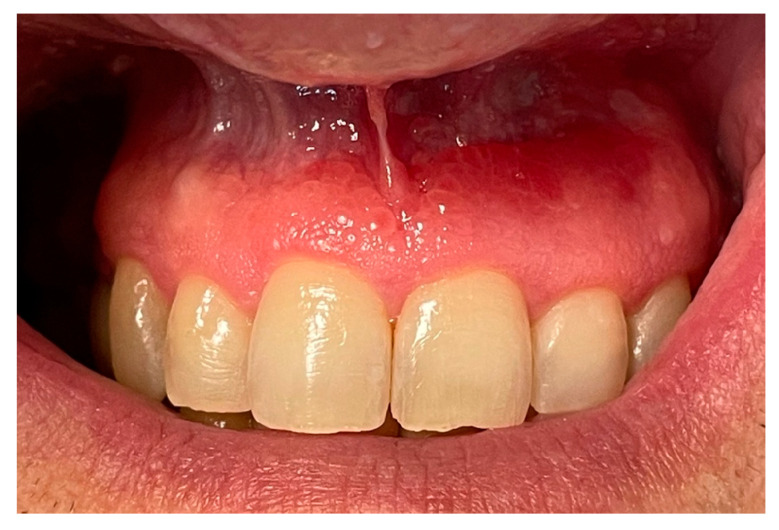
Four-week follow-up image showing partial remission of the lesion: decreased erythema and edema in the keratinized gingiva.

The patient underwent multiple follow-up visits. At four weeks, the lesion appeared to be in remission (Figure 4), with a marked reduction in erythema and edema of the keratinized gingiva. However, involvement of the alveolar mucosa persisted.

After a drug-free interval of two weeks, a second identical two-week treatment cycle was prescribed due to the persistence of mild residual clinical signs.

**Figure 5:**
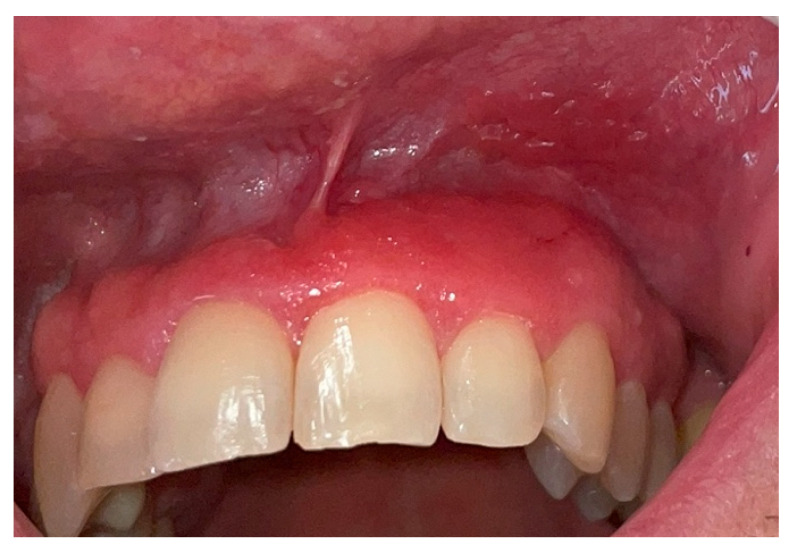
Clinical status at eight-week follow-up. The lesion appears stable with residual mucosal involvement.

At the eight-week follow-up visit, a minor residual lesion was still present, particularly in the alveolar mucosa, although the patient reported complete resolution of the symptoms experienced prior to treatment initiation (Figure 5).

## 3. Discussion

The etiology of PCG is unclear: the main etiologic factors could be allergenic reactions associated with cinnamaldehyde, cinnamon, different herbal constituents of toothpaste as reported by Macleod et al. in 1989 [11] and Anil in 2007 [12], and khat leaves as reported in a study by Marker et al. [13].

The analysis of the available scientific evidence indicated a female predominance, with a male-to-female ratio of 1:1.42. Reported cases ranged in age from 12 to 70 years, with a mean age of 31.6 years, while occurrence in the pediatric population was rare. Lesions were more frequently observed in the maxilla, predominantly affecting the anterior gingiva [7].

Diagnosis of PCG was based on intraoral findings characterized by an erythematous gingiva with loss of stippling [14]. First oral examinations performed and described by Leuci et al. [1] allowed clinicians to detect lesion characteristics in 40 out of 45 cases, specifying the predominant clinical phenotype or provisional diagnosis for each. Among these groups, 25 cases (62.5%) were identified as bullous or nonspecific blistering mucositis, 4 cases (10%) were classified as erythematous lesions consistent with erythroplakia, 4 cases (10%) as keratotic lesions resembling oral lichenoid conditions, 4 cases (10%) as verruciform lesions with features suggestive of malignancy, and 3 cases (7.5%) as ulcerative lesions of indeterminate etiology [1]. In this case report, the lesion appeared polypoid, erythematous, and edematous at the baseline.

The gold standard for definitive diagnosis of PCG is histopathology, which facilitates its differentiation from other entities presenting with similar clinical characteristics [15]. The clinical differential diagnosis should consider various forms of desquamative gingivitis, including erythematous or erosive lichen planus, as well as autoimmune vesiculobullous disorders such as mucous membrane pemphigoid and pemphigus vulgaris. Additionally, gingival conditions associated with hormonal changes, such as pubertal or pregnancy-induced gingivitis, and systemic diseases like leukemia-related gingivitis should also be taken into account [16].

In the study of Negi et al. [17], histopathological analysis was performed on an incisional biopsy obtained from the gingiva. Microscopic examination revealed fragments of tissue lined by hyperplastic parakeratinized stratified squamous epithelium. The underlying connective tissue exhibited a dense chronic inflammatory infiltrate, predominantly composed of plasma cells, with numerous and diffused endothelium-lined vascular channels containing erythrocytes, all set within a moderately collagenous stroma. To confirm the plasma cell population, immunohistochemical staining for kappa and lambda light chains was conducted. The results showed strong positivity for both kappa and lambda light chains, indicating a polyclonal plasma cell infiltrate. Based on these findings, a definitive diagnosis of PCG was made [17]. Following the above-cited study, the current immunohistochemical investigation revealed the presence of a dense plasma cell infiltrate characterized by a liquenoid pattern, comprising CD20-positive B lymphocytes and CD3-positive T lymphocytes. Additionally, a population of plasma cells was identified, showing expression of both lambda and kappa light chains of immunoglobulins.

Furthermore, hematologic investigations may be conducted to exclude systemic blood disorders requiring a different therapeutic approach [18]. For this reason, in the present case, the hematochemical laboratory tests played a crucial role in the diagnostic process, confirming the solitary nature of the lesion within the oral cavity and excluding any systemic involvement.

Regarding treatment, the first-line strategy involves identifying and eliminating potential etiologic factors, as PCG is categorized under hypersensitivity reactions. Atarbashi-Moghadam et al. [4] reported that possible allergens were identified in 47% of cases; however, only 44.44% of these showed complete remission following allergen removal. In the remaining cases, symptom persistence suggests the presence of additional contributing factors. Topical pharmacologic treatments, including corticosteroids (e.g., triamcinolone acetate 0.1%, hydrocortisone 0.5%, clobetasol 0.05%, fluocinonide 0.05%), antihistamines, and steroid antibiotics (e.g., fusidic acid 2%), have demonstrated inconsistent results. Remission was observed in isolated cases, often requiring combination therapy [4]. Side effects such as fungal infections and lesion recurrence were reported. Surgical excision of lesions, through gingivectomy or biopsy, was the most effective treatment, with complete remission achieved in 88.88% of the cases. Photobiomodulation (PBM) has shown positive results in a single reported case, exhibiting anti-inflammatory and immunosuppressive effects. It may be considered a viable treatment option due to its minimal adverse effects, particularly in patients who do not respond to conventional therapies or in recurrent cases [4]. In this case report, the therapy consisted of a compound of clobetasol propionate 0.05% and hydroxypropyl methylcellulose oral base (Biotene Gel) to enhance the adhesion and long-lasting effect of the topical corticosteroid on the oral mucosa.

Long-term reliance on corticosteroids is problematic, as chronic steroid use carries well-known risks, such as increased risk of infections, osteoporosis, glaucoma, and depression. Natural polyphenolic compounds have been proposed as potential adjuncts due to their anti-inflammatory properties and favorable safety profile. Although not yet standard treatment, they represent a promising direction for future therapeutic strategies in chronic oral inflammation [19].

In our case report, the treatment led to symptom improvement without achieving complete clinical resolution of the lesion. Similar results have been documented by Joshi et al. [18], while Leuci et al. [1] reported complete remission in only 10% of cases. These findings suggest that, although therapy can markedly improve patient comfort in PCG, it may not always result in total lesion resolution.

A primary limitation of this study lies in its design as a single-patient case report, which represents a low level of scientific evidence and does not allow for broad generalization of the findings. Another limitation is the incomplete clinical resolution, which may be attributed to the inability to identify a specific causative antigen and to fully elucidate other contributing pathogenic factors. It is also noteworthy that the involvement of the alveolar mucosa persisted, a region where the steroid gel’s retention might have been less effective.

## 4. Conclusions

Plasma cell gingivitis remains a rare inflammatory condition of the gingival tissues. Future studies should focus on enhancing early diagnostic accuracy by developing more sensitive and specific clinical and histopathological markers.

Innovative targeted treatment protocols that combine topical and systemic approaches, potentially including immunomodulatory agents, should be explored. These strategies should aim not only to achieve complete clinical remission but also to minimize recurrence and enhance long-term patient outcomes.

## Figures and Tables

**Table 1:** Results of hematological and inflammatory profile, including complete blood count with differential, reticulocyte count, erythrocyte sedimentation rate (ESR), and quantitative C-reactive protein (CRP) levels.

Parameter	Result	Unit	Reference Range
White Blood Cells (WBC)	6.89	10^3^/µL	4.00–10.00
Neutrophils (Neu#)	4.21	10^3^/µL	1.50–8.00
Lymphocytes (Lymph#)	1.52	10^3^/µL	0.80–4.00
Monocytes (Mono#)	0.61	10^3^/µL	0.10–1.00
Eosinophils (Eos#)	0.52	10^3^/µL	0.00–0.70
Basophils (Bas#)	0.03	10^3^/µL	0.00–0.20
Neutrophils (%) (Neu%)	61.0	%	40.0–74.0
Lymphocytes (%) (Lymph%)	22.1	%	20.0–45.0
Monocytes (%) (Mono%)	8.9	%	3.0–11.0
Eosinophils (%) (Eos%)	7.5	%	0.0–7.0
Basophils (%) (Bas%)	0.5	%	0.0–1.5
Red Blood Cells (RBC)	4.65	10^6^/µL	4.40–5.80
Hemoglobin (Hb)	14.9	g/dL	13.0–17.0
Hematocrit (HCT)	44.7	%	40.0–52.0
Platelets (PLT)	376	10^3^/µL	130–450
Reticulocyte Count (RET#)	58.6	10^9^/µL	20.0–200.0
Immature Reticulocyte Fraction (IRF)	7.5	%	0.0–25.0
Erythrocyte Sedimentation Rate (ESR)	3	mm	3.0–15.0
C-Reactive Protein (Quantitative) *	1.11	mg/L	0–5.0

* Method: immunometric. The symbol # stands for absolute cell count, as distinct from the columns with the symbol %, which show the relative percentages.

## Data Availability

Data will be made available by the corresponding author upon reasonable request.
